# Salicylic acid delays pear fruit senescence by playing an antagonistic role toward ethylene, auxin, and glucose in regulating the expression of *PpEIN3a*

**DOI:** 10.3389/fpls.2022.1096645

**Published:** 2023-01-11

**Authors:** Yue Xu, Liyue Huo, Keke Zhao, Yawei Li, Xinran Zhao, Huiying Wang, Wenli Wang, Haiyan Shi

**Affiliations:** College of Horticulture, Hebei Agricultural University, Baoding, Hebei, China

**Keywords:** pear (*Pyrus pyrifolia* Nakai. ‘Whangkeumbae’), fruit senescence, salicylic acid, ethylene insensitive3(EIN3), auxin, glucose

## Abstract

Salicylic acid (SA) and ethylene (ET) are crucial fruit senescence hormones. SA inhibited ET biosynthesis. However, the mechanism of SA delaying fruit senescence is less known. ETHYLENE INSENSITIVE 3 (EIN3), a key positive switch in ET perception, functions as a transcriptional activator and binds to the primary ET response element that is present in the promoter of the *ETHYLENE RESPONSE FACTOR1* gene. In this study, a gene encoding putative EIN3 protein was cloned from sand pear and designated as *PpEIN3a*. The deduced PpEIN3a contains a conserved EIN3 domain. The evolutionary analysis results indicated that PpEIN3a belonged to the EIN3 superfamily. Real-time quantitative PCR analysis revealed that the accumulation of *PpEIN3a* transcripts were detected in all tissues of this pear. Moreover, *PpEIN3a* expression was regulated during fruit development. Interestingly, the expression of *PpEIN3a* was downregulated by SA but upregulated by ET, auxin, and glucose. Additionally, the contents of free and conjugated SA were higher than those of the control after SA treatment. While the content of ET and auxin (indole-3-acetic acid, IAA) dramatically decreased after SA treatment compared with control during fruit senescence. The content of glucose increased when fruit were treated by SA for 12 h and then there were no differences between SA treatment and control fruit during the shelf life. SA also delayed the decrease in sand pear (*Pyrus pyrifolia* Nakai. ‘Whangkeumbae’) fruit firmness. The soluble solid content remained relatively stable between the SA treated and control fruits. This study showed that SA plays an antagonistic role toward ET, auxin, and glucose in regulating the expression of *PpEIN3a* to delay fruit senescence.

## 1 Introduction

Pear, a kind of popular fruit crop, has been cultivated around the world (Gu et al., 2020). According to the statistics of the Food and Agriculture Organization of the United Nations (FAOSTAT), planting area of pear in the world has 1.29 million hectares and its global production is 23.1 million tons in 2020 (http://data.un.org/). Climacteric fleshy fruit are known to go through an ethylene respiratory burst at the beginning of their ripening process (McMurchie et al., 1972; Osorio et al., 2013). Pear is a typical climacteric fruit, which has many important physiological changes after ripening. The ripening of pear is usually characterized by increases in sugar and ethylene content as well as changes in fruit color and firmness (Zhang et al., 2020a). ‘Whangkeumbae’ is one variety of sand pear (*Pyrus pyrifolia*). Its fruit is known for its smooth surface and good flavor. However, the shelf life of ‘Whangkeumbae’ fruit is short due to postharvest ethylene outbreaks and diseases. ‘Whangkeumbae’ pear (*Pyrus pyrifolia*) is a climacteric fruit and experiences a typical burst of ET production at 10 d after harvest (Shi et al., 2013).

Ethylene plays an important role in fruit ripening (Yue et al., 2020). The members of the ETHYLENE INSENSITIVE3/ETHYLENE INSENSITIVE3-LIKE1 (EIN3/EIL1) (Chao et al., 1997; Guo and Ecker, 2003; Potuschak et al., 2003; Gagne et al., 2004) family are transcription factors and the crucial regulators of ET signaling that maintain various plant responses to ET (Dolgikh et al., 2019), including fruit ripening (Burg and Burg, 1962; Theologis et al., 1992; Zhu et al., 2022), leaf senescence (Gepstein and Thimann, 1981; Li et al., 2013), and resistance to pathogen infections (Alonso et al., 2003; Chen et al., 2009). In these modulations, the transcriptional control that is conferred by the transcription factor EIN3 is pivotal (Boutrot et al., 2010). EIN3, a key positive switch in ethylene perception, functions as a transcriptional activator and binds to the primary ethylene response element that is present in the promoter of the *ETHYLENE RESPONSE FACTOR1* gene (Chao et al., 1997; Solano et al., 1998). EIN3 transcriptionally activates the expression of *ERF1*, which has the core sequence 5’-ATGTA-3’ within its promoter (Solano et al., 1998; Chen et al., 2009; Zhong et al., 2009; Boutrot et al., 2010). EIL and ERF, as transcriptional complexes, coordinate to regulate the expressions of ripening-related genes during the ripening process of kiwifruit (Yin et al., 2010).

EIN3 is degraded *via* the ubiquitin/26S proteasome in the absence of ethylene. However, EIN3 is quickly stabilized and accumulated in the nuclei under ethylene treatment (Guo and Ecker, 2003; Potuschak et al., 2003; Yanagisawa et al., 2003; Gagne et al., 2004). Zhao et al. (2021) showed that PuEIN3 interacts with PuEBF1 and PuEBF2 at the protein level in *Pyrus ussuriensis* cv. Nanguo, suggesting that it may be regulated by ubiquitination pathway. In addition to EIN3, five EIL transcription factors (e.g., EIL1 to EIL5) have been identified from *Arabidopsis* that may also contribute to ethylene signaling (Chao et al., 1997; Wang et al., 2002). Zhang et al. (2020a) reported methyl salicylate (MeSA) decreased the expression of *PbACS4*, *PbACO1*, *PbACO4*, *PbETR1*, *PbETR2*, *PbERS2*, *PbCTR1*, *PbEIN2* and *PbEIL1* genes related to ethylene biosynthesis and perception, and enhanced the expression of ERS1 receptor genes, suggesting that MeSA regulates fruit ripening and senescence by directly affecting ethylene response. A recent study showed that EIN3 physically interacted with the core SA signaling regulator, NPR1, in senescing leaves. Additionally, EIN3 and NPR1 synergistically upregulated the expressions of senescence-associated genes (e.g., *ORE1* and *SAG29*) (Wang et al., 2021).

Salicylic acid (SA) is an important plant hormone that has been shown to delay flower and fruit senescence (Hassan et al., 2007; Dokhanieh et al., 2013; Shabanian et al., 2019). Ethylene, a plant hormone, plays an important role in the ripening process of climacteric fruit (Lelièvre et al., 1997; Nath et al., 2006). Several recent studies have also revealed that SA and ethylene (ET) promoted leaf senescence through a coordinated series of molecular and physiological events (Wang et al., 2021; Yu et al., 2021). Additionally, SA delayed banana (Srivastava and Dwivedi, 2000) and sweet cherry (Valero et al., 2011) fruit ripening. Because SA can inhibit ET biosynthesis from 1-aminocyclopropane-1-carboxylic (ACC) (Leslie and Romani, 1988) through suppressing ACC oxidase activity (Fan et al., 1996). Recently, SA has been reported to regulate the expressions of several genes that are involved in ET biosynthesis and signaling pathways (Shi and Zhang, 2012; Shi et al., 2013; Zhang et al., 2013; Shi et al., 2019; Shi et al., 2021). High concentrations of indole-3-acetic acid (IAA) induced *PpACS1* and *PpACO1* gene expression, resulting in the production of large amounts of ethylene. Low content of IAA in the late ripening stage of stony hard peaches led to few ethylene yield and inhibited fruit softening (Tatsuki et al., 2013). Naphthalene acetic acid (NAA) can promote ethylene production in *Prunus salicina* (El-Sharkawy et al., 2014). Yue et al. (2020) showed auxin induced ET biosynthesis in apple fruit through activation of *MdARF5* expression. In sand pear, IAA can induce the expression of *PpACS1a* (Shi and Zhang, 2014). Yanagisawa et al. (2003) found that glucose enhances the degradation of EIN3, a key transcriptional regulator in ethylene signaling, through the plant glucose sensor hexokinase8. Ethylene, by contrast, enhanced the stability of EIN3. The *ein3* mutant had a glo phenotype, and overexpression of EIN3 in transgenic Arabidopsis decreased glucose sensitivity. Our previous study demonstrated that the expression of *PpEIN3b* was repressed by SA and glucose, but was induced by ACC during sand pear fruit ripening and senescence (Shi et al., 2019). However, the molecular mechanism by which SA, ET, auxin, and glucose interact to regulate fruit senescence is still unclear. This study aims to elucidate the mechanism by which SA delays fruit senescence by regulating the expression of *PpEIN3a* under SA/ET/auxin/glucose treatments, which would provide valuable information for regulating sand pear fruit senescence.

## 2 Materials and methods

### 2.1 Collection of plant materials

Sand pear (*Pyrus pyrifolia* Nakai. ‘Whangkeumbae’) fruit were collected as described in the study (Shi et al., 2019) from Hebei Agricultural University (Baoding, China). The pulp of these pears was collected for further study. Shoots were collected at 5 days old, young stems were collected at 10 days old, and young leaves were collected at 5 days old. Age (day old) was calculated from the starting time when buds that had just emerged in pear trees could be observed by the naked eye. All samples including petals and anthers were prepared as described in Shi et al. (2019).

### 2.2 Fruit treatments

The pear fruit with uniform size, free from mechanical damage, diseases and pests were screened at 150 days after full bloom (DAFB) and stored at 4°C for two months and then transferred to shelf life at room temperature for 36 h. Fruits were divided into two groups. One group was soaked in 200 μM SA (Solarbio, Beijing, China) for 12 h. The second group was soaked in distilled water for 12 h as control. The SA-treated and control fruit were air-dried and stored at room temperature for 12 h and 24 h. All fruit were stored at room temperature for 36 h and sampled every 12 h. For each sampling point, three biological replicates with five fruit each were used for analysis.

Pulp samples at one week after fruit harvest were separately soaked in 2, 20, 200, and 2000 μM SA (Solarbio, Beijing, China) for 12 h at room temperature, and the control was soaked in distilled water for 12 h (Shi et al., 2019). One week after harvest, fruit pulp samples were soaked in 1 μL/L ethephon (Solarbio, Beijing, China; Zhang et al., 2013) and 200 μM IAA (Solarbio, Beijing, China) for 6, 12, and 24 h (Shi and Zhang, 2014). Concurrently, the controls of the ethephon and IAA treatments were soaked in distilled water for 6, 12, and 24 h, respectively. The pear fruit pulp was treated with 15% glucose (Solarbio, Beijing, China; Shi et al., 2019) for 6, 12, and 24 h. The pulp was soaked concurrently in ddH_2_O for 6, 12, and 24 h as corresponding controls. All samples were frozen as described in the previous study (Shi et al., 2019).

### 2.3 Isolation of *PpEIN3a* cDNA

Total RNA was extracted from pear fruit that were collected at 150 d after full bloom. Complementary DNA (cDNA) library was constructed as described in primary study (Shi and Zhang, 2012) for sequencing. One clone that encoded EIN3 was identified and designated as *PpEIN3a*.

### 2.4 RNA isolation and real-time quantitative PCR analysis

Total RNA was isolated from the shoots, young stems, young leaves, petals, anthers, developing pear pulp, and pulp that was treated with SA/ethephon/IAA/glucose by a previously described method (Li et al., 2005).

The *PpEIN3a* expression profiling was performed using real-time quantitative PCR *via* a Magic SYBR mixture according to the manufacturer’s instructions (CoWin Biosciences, China) in the detection system (Mastercycler ep realplex 4, Eppendorf AG, Hamburg, Germany). A pear *actin* gene was used as a standard control for the real-time quantitative PCRs. A two-step RT-PCR procedure was carried out in all experiments *via* a previously described method (Li et al., 2005). The gene-specific primer sequences for *PpEIN3a* were 5’-GGAGTTGATGATGGGCAGAAAATG-3’ and 5’-GGTTCAGACATGTTGATGTTGCAT-3’. The relative value for the expression level of *PpEIN3a* gene was calculated by Y=10^DCt/3.75^*100% (DCt is the differences of Ct between the control *actin* products and the target *PpEIN3a* products, i.e., DCt=Ct*_PpEIN3a_*-Ct*actin*, and 3.75 is a parameter for the cycle number which represents a 10-fold expression difference between the target gene and control gene).

### 2.5 DNA sequencing and protein analyses

The sequence analyses of the isolated pear *EIN3*a gene (cDNA) and its deduced protein were done with DNA STAR software (DNA STAR Inc., Madison, WI, USA). The conserved domain was determined by National Center for Biotechnology Information (NCBI) Conserved Domain Search (http://blast.ncbi.nlm.nih.gov/Blast.cgi). The protein multiple sequence alignment analysis was performed with Clustal Omega (https://www.ebi.ac.uk/Tools/msa/clustalo/), and protein motif analysis was performed using Motif Scan (http://myhits.isb-sib.ch/cgi-bin/motif_scan). The evolutionary relationships among twenty-nine EIN3 protein sequences from different plants were determined by MEGA7 software (Kumar et al., 2016) based on the neighbor-joining method (Saitou and Nei, 1987). The genomic DNA sequence of *PpEIN3a* was analyzed according to genome information of sand pear (Gao et al., 2021) by using the GDR database (https://www.rosaceae.org/).

### 2.6 Extraction and determination of SA content

High performance liquid chromatography (HPLC) was used to determine the content of SA (Zhang et al., 2003). The frozen sand pear pulp was ground into good powder in liquid nitrogen, and for the extraction of SA, a 0.8 g sample was soaked with 1 mL 90% (v/v) methyl alcohol in the dark for 12 h at 4°C. Then, the sample was centrifuged at 8,000 *g* for 10 min at 4°C. The supernatant was transferred into a clean 10-mL centrifugal tube and stored for 2 h at 4°C. The remaining sediment was re-suspended in 0.5 mL 90% methyl alcohol and re-extracted for 2 h at 4°C. The above sample was centrifuged again. The two resulting supernatants were mixed together and dried with N_2_ to approximately 0.3 mL aqueous solution. Then, 20 μL of 1 mg/mL trichloroacetic acid was added. After shaking, 1 mL cyclohexane and ethyl acetate mixture (V/V=1/1) was added for extraction, which was repeated twice. The upper organic phase was transferred to a clean 10-mL centrifugal tube and dried with N_2_. A 0.5 mL methyl alcohol was added to dissolve and filter, and the obtained solution represented the free SA in the sample.

A 0.5 mL of 2 M HCl was added into the remaining aqueous phase. After shaking, the mixture was heated in 80°C-water bath for 1 h and, after cooling, was extracted twice with cyclohexane and ethyl acetate (V/V=1/1). The rest procedure is the same as above, and the obtained solution represented the conjugated SA in the sample.

The contents of free SA and conjugated SA in sand pear pulp were determined by high performance liquid chromatography (HPLC, Agilent 1260, USA). Agilent Eclipse Plus C18 column (250 mm × 4.6 mm, 5 μm; Agilent, USA) was used to elute the SA with 65% acetonitrile and methyl alcohol. The column temperature was 25°C, 20 μL of sample was used for detection, the flow rate was set at 1 mL/min, and the detection wavelength was 280 nm.

Each experiment was repeated independently three times, and the data were analyzed using GraphPad Prism 8. Independent *t* tests for equality of means showed very significant differences (** *p* value < 0.01) between the controls and treated fruit.

### 2.7 Determination of ethylene production

A 2 g frozen sand pear pulp was ground into powder with liquid nitrogen. It was transferred to a clean 10-mL tube and added 4 mL of 95% ethanol. The mixture was boiled and extracted for 20 min. After cooling, the mixture was centrifuged for 15 min at 10,000 *g*. Then, the supernatant was transferred into a clean 10-mL tube. The remaining sediment was re-suspended with 4 mL 80% ethanol and re-extracted for 30 min at 70°C. The above sample was centrifuged again. Two supernatants were merged together and evaporated to dry by a rotary evaporator (IKA RV10, GER) at 40°C. Then 2 mL of distilled water was added to evaporated residue. The evaporated residue was shaken and dissolved. The preparation solution was obtained and stored at −20°C for further analysis (Lizarda and Yang, 1979).

The ethylene production in frozen samples was detected following the methods of Fan et al. (1998) with some modifications. A 1 mL of the preparation solution was taken and put into a 10 mL sample bottle. Then 40 μL of 25 mM/L HgCl_2_ was added into the bottle. The reaction sample bottles were then sealed with rubber stopper and kept in ice for 10 min. Then 0.2 mL of precooled mixture of 5% NaClO and NaOH (V/V=2/1) was injected into the reaction vessel by l-mL syringe. The mixture was shaken rapidly for 5 s and put into the ice for 5 min. A 1 mL of gas from the headspace was taken to measure the ethylene production by a gas chromatography (Agilent 7820A, USA) with an FID detector. Agilent GS-Alumina column (30 m × 0.53 mm) was used to elute the ethylene production. The column temperature was 80°C and the injection volume was 1 mL (Lizarda and Yang, 1979).

### 2.8 Extraction and measurement of IAA content

The method of Chen et al. (2003) for extraction of IAA was modified. A 2 g frozen sand pear pulp was ground into good power with liquid nitrogen. A 2 mL of pre-cooled 80% methyl alcohol was added into the power and the sample was transferred to a clean 10-mL tube. Following the remaining power was washed with 2 mL of pre-cooled 80% methyl alcohol and transferred to the clean 10-mL tube, which repeated twice. The mixture in the 10-mL tube was added with 500 μL of butylated hydroxytoluene (BTH) and soaked in the dark for 12 h at 4°C. In dark, the samples were shaken by ultrasonication and vortexing (Kunshan Schwimmer ultrasonic cleaner, KQ-100DE, China) for 1 h, and centrifuged at 12,000 *g* for 15 min at 4°C. The supernatant was transferred into a clean 10-mL tube and dried with N_2_ to 1-2 mL aqueous solution, and the pH was adjusted to 8.0 by 1 M dipotassium hydrogen phosphate and 1% acetic acid. Following 0.4 g polyvinylpyrrolidone (PVPP) was added to remove impurities such as phenols. The mixture was shaken for 0.5 h by ultrasonication and then centrifuged at 12,000 *g* for 5 min at 4°C. The supernatant was transferred into a clean centrifuge tube, and the pH was adjusted to 3.0 *via* 1 M citric acid and 1% NaOH. Following 3 mL ethyl acetate was added into the aqueous phase, shaken for 0.5 h, and placed for 2 h at 4°C. The upper solution was transferred to a clean 10-mL tube and dried with N_2_.

A C18 solid phase column was activated by adding, successively, 4 mL of methyl alcohol and 2 mL of sterile water. A 2 mL of 20% methyl alcohol was added into the 10-mL tube to dissolve the purified and dried supernatant. The mixture was poured into the activated C18 column. Following the column was washed with 2 mL of 10% methyl alcohol. Finally, 1 mL of pH 8 methyl alcohol was used to elute IAA from the column and through a 0.22 μm filter.

The determination of IAA was performed by HPLC (Agilent 1260, USA) and the C18 column (250 mm × 4.6 mm, 5 μm; Agilent, USA) was used to elute the IAA. Mobile phase A was sterile water, mobile phase B was acetonitrile, mobile phase C was 0.7% acetic acid, and mobile phase D was methyl alcohol. The column temperature was 30°C, 10 μL of sample was used for detection, the flow rate was set at 0.7 mL/min, and the detection wavelength was 254 nm.

Each experiment was repeated independently three times, and the data were analyzed using GraphPad Prism 8. Independent *t* tests for equality of means showed very significant differences (** *p* value < 0.01) between the controls and treated fruit.

### 2.9 Determination of glucose content

The frozen sand pear pulp was ground into homogenate with 20 mL sterile water for determination of glucose content. A 0.5 g sample was transferred to a clean conical bottle and was heated in a 70-80°C water bath for 3 h. After cooling, the sample was centrifuged at 8,000 *g* for 10 min. Then, the supernatant was transferred into a clean 10-mL tube. A 1 mL of the final supernatant was filtered through a 0.22 μm filter before analyses (Wang, 2013).

The measurement of glucose content was performed by HPLC (Agilent 1260, USA) and chromatographic column (Shodex Asahipak NH2P-50 4E, 4.6 mm × 250 mm, 5 μm) was used to elute the glucose. Solvent was 25% sterile water and 75% acetonitrile. The flow rate was set at 0.6 mL/min, the column temperature was 32°C, and 5 μL of sample was used for detection.

### 2.10 Determination of firmness and soluble solids contents

The fruit were characterized after the 200 μM SA treatment by measurements of fruit firmness and soluble solids contents (SSC) in fruit samples. Firmness was measured on both sides of the fruit after peeling, using a hardness tester (GY-1, China) that was fitted with a 7.5 mm diameter probe at a rate of 10 mm/s. The data were recorded as kg-force and converted to Newtons (Huang et al., 2021). The SSCs of fruit were determined from juice samples from both sides of the fruit after peeling with a digital refractometer (“Pocket” PAL-1, 0-53%, Atago, Japan). Each experiment was repeated independently three times, and the figures were made with GraphPad Prism 8. Independent *t* tests for equality of means showed significant differences (*p value < 0.05) or very significant differences (** *p* value < 0.01) between the controls and treated fruit.

After measurement of firmness and SSC content, fruit slices of pulp without skin from same batch of fruit were frozen in liquid nitrogen and stored at −80°C until determination of hormone and glucose contents.

### 2.11 Statistical analysis

Each experiment was repeated independently three times, and the data were analyzed using SPSS. Independent *t* tests for equality of means showed significant difference (*p value < 0.05) or very significant difference (**p value < 0.01) between the control and treated fruit.

## 3 Results

### 3.1 Cloning and characterization of *PpEIN3a*

**Figure 1:**
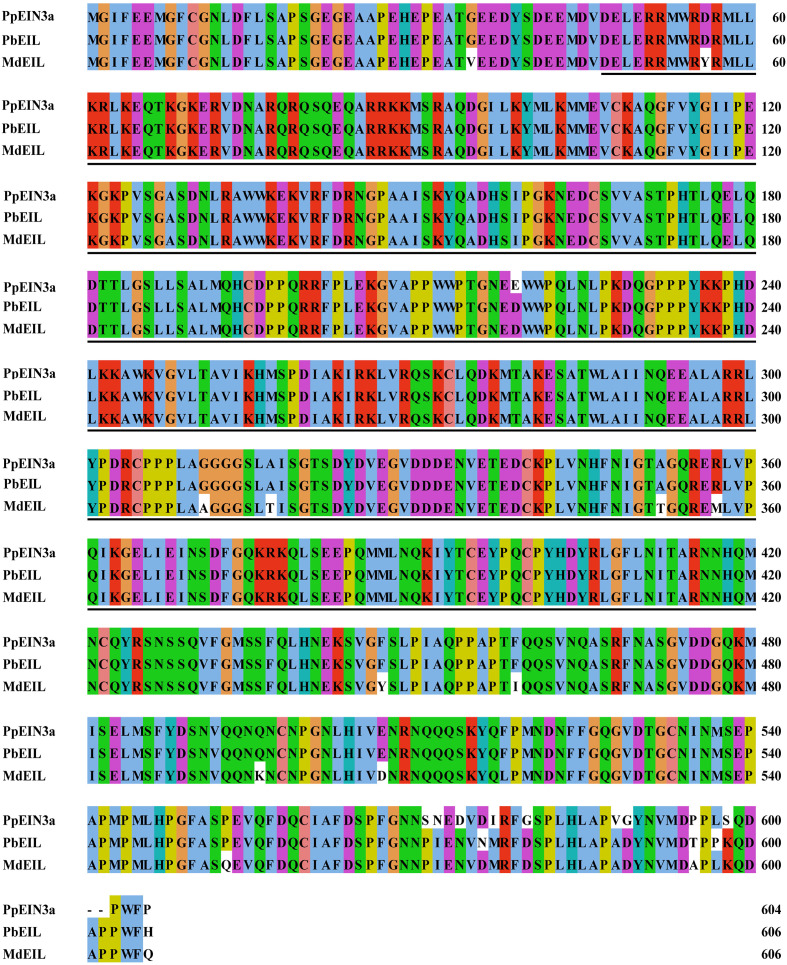
Sequence alignment among the three fruit EIN3/EIL proteins. The sequences of fruit EIN3/EILs were aligned. Amino acid substitutions and deletions are highlighted in white. The ethylene insensitive 3 (EIN3) domains are shown in underlines. The accession numbers of these fruit EIN3/EIL proteins in GenBank are KT726838 (*Pyrus pyrifolia* PpEIN3a), XP_009355060 (*Pyrus x bretschneideri* PbEIL), and XP_008346133 (*Malus x domestica* MdEIL).

One cDNA encoding the EIN3 protein was identified by screening the sand pear fruit cDNA pool (Shi and Zhang, 2012). The cloned cDNA was designated as *PpEIN3a*, accession number in GenBank: KT726838. The genomic DNA sequence of *PpEIN3a* was analyzed according to sand pear genome database (Gao et al., 2021). *PpEIN3a* cDNA encodes an EIN3 homolog that consists of 604 amino acids and shares relatively high homology with pear PbEIL (*Pyrus x bretschneideri*, XP_009355060; 97.52% identity) and PuEIL (*Pyrus ussuriensis x Pyrus communis*, KAB2627254; 97.52% identity) at the amino acid level. The PpEIN3a protein contains a conserved ethylene insensitive 3 (EIN3) domain (**Figure 1**). All three sequences contain a conserved ethylene insensitive 3 (EIN3) domain, which suggests that PpEIN3a may have similar function with PbEIL and PuEIL.

As shown in **Figure 1**, twelve substitutions and two deletions at the amino acid level occurred in PpEIN3a compared with other fruit EIN proteins. Among these, two substitutions may be crucial for the function of PpEIN3a. Namely, the nonpolar Pro was replaced by a polar Ser at position AA571. At position AA572, the nonpolar Ile was substituted by a polar Asn. Moreover, at positions AA599 and AA600, PpEIN3a has deleted the two amino acids (e.g., Asp and Ala).

### 3.2 Evolutionary relationships of PpEIN3a with other EIN3/EIL proteins

**Figure 2:**
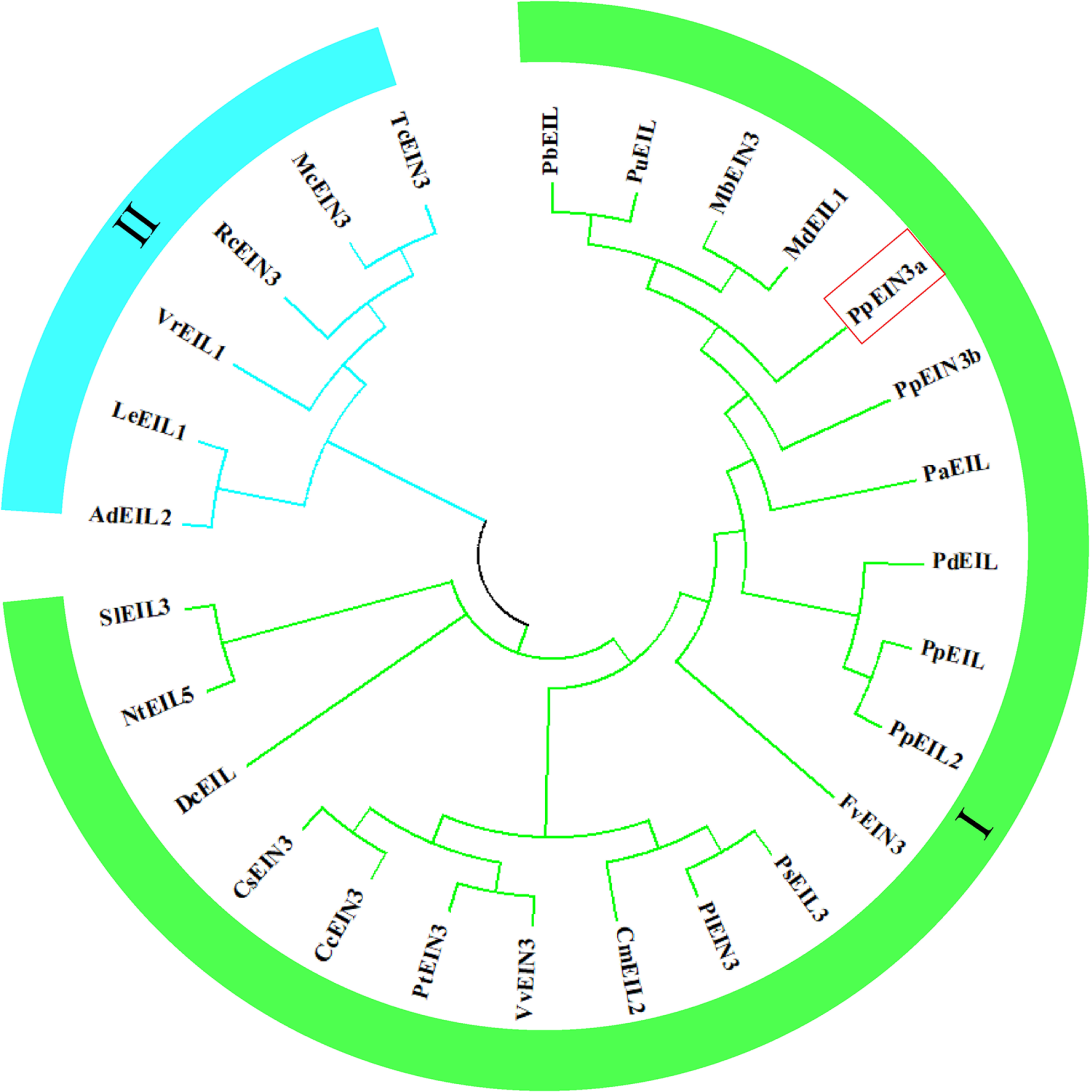
Evolutionary relationships of the PpEIN3a protein with respect to other EIN3/EIL proteins. The neighbor-joining evolution tree was constructed in MEGA 7. The accession numbers of the plant EIN3/EIL proteins in GenBank are: KT726838 (*Pyrus pyrifolia* PpEIN3a), XP_009355060 (*Pyrus x bretschneideri* PbEIL), KAB2627254 (*Pyrus ussuriensis x Pyrus communis* PuEIL), TQD73050 (*Malus baccata* MbEIN3), XP_008346133 (*Malus x domestica* MdEIL1), KT726839 (*Pyrus pyrifolia* PpEIN3b), XP_034202621 (*Prunus dulcis* PdEIL), XP_020411970 (*Prunus persica* PpEIL), XP_021834590 (*Prunus avium* PaEIL), EF031066 (*Prunus persica* PpEIL2), XM_006379360 (*Populus trichocarpa* PtEIN3), NM_001247617 (*Solanum lycopersicum* SlEIL3), XM_002530146 (*Ricinus communis* RcEIN3), XM_006432473 (*Citrus clementina* CcEIN3), XM_006471230 (*Citrus sinensis* CsEIN3), XM_002276344 (*Vitis vinifera* VvEIN3), AB525913 (*Daucus carota* DcEIL), XM_004306392 (*Fragaria vesca* FvEIN3), EU887511 (*Actinidia deliciosa* AdEIL2), XM_007016620 (*Theobroma cacao* TcEIN3), JQ771471 (*Paeonia suffruticosa* PsEIL3), JX445144 (*Paeonia lactiflora* PlEIN3), FJ890314 (*Lithospermum erythrorhizon* LeEIL1), AB063192 (*Cucumis melo* CmEIL2), AY248907 (*Nicotiana tabacum* NtEIL5), AF467784 (*Vigna radiate* VrEIL1), and KF595122 (*Momordica charantia* McEIN3).

The analysis of the evolutionary relationships among PpEIN3a and other EIN3/EIL proteins, which was previously reported in plants, is shown in **Figure 2**. The evolutionary tree was divided into two subgroups. The PpEIN3a protein has the closest evolutionary relationship with pear PbEIL (*Pyrus x bretschneideri*, XP_009355060); pear PuEIL (*Pyrus ussuriensis x Pyrus communis*, KAB2627254); wild apple MbEIN3 (*Malus baccata*, TQD73050) (Chen et al., 2019); and apple MdEIL1 (*Malus domestica*, XP_008346133) among all EIN3/EIL proteins. PpEIN3a also has a close evolutionary relationship with sand pear PpEIN3b (*Pyrus pyrifolia*, KT726839) (Shi et al., 2019). However, PpEIN3a has relatively distant evolutionary relationships with the six EIN3/EILs that occupy another clade of the tree (subgroup II, **Figure 2**). These results suggest that PpEIN3a might have diverged earlier from these EIN3s during evolution.

### 3.3 Expression profiling of the *PpEIN3a* gene in sand pear

**Figure 3:**
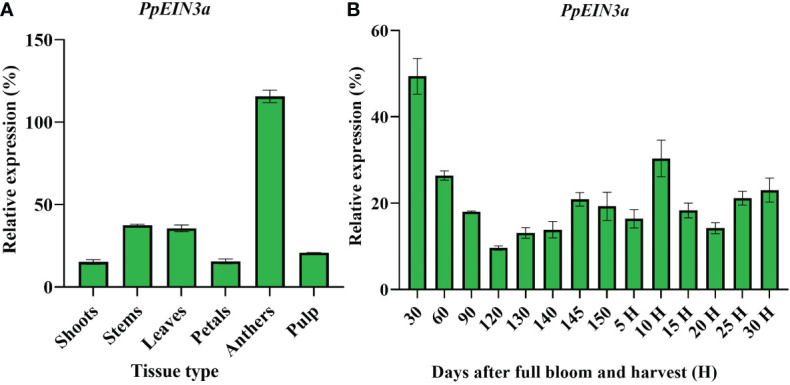
Real-time quantitative PCR analyses of *PpEIN3a* expressions in different pear tissues **(A)** and during fruit development **(B)**. Total RNA was isolated from different tissues (e.g., shoots, young stems, young leaves, petals, anthers, and pulp) and during fruit development. The relative value of the *PpEIN3a* expression in pear is shown as a percentage of *actin* expression activity. The mean values and SD (bar) from three independent experiments are shown. The values shown are the means ± SD for three replicates.

Real-time quantitative PCR analysis was performed to explore the *PpEIN3a* gene expression pattern. *PpEIN3a* was preferentially expressed in sand pear anthers. It exhibited a moderate expression activity in young stems and young leaves. A relatively weak expression signal of *PpEIN3a* was detected in the pulp, shoots, and petals (**Figure 3A**).

Further analysis of the expression pattern of *PpEIN3a* during fruit development was performed to investigate whether the expression of *PpEIN3a* is regulated during fruit development. The experimental results (**Figure 3B**) revealed that *PpEIN3a* exhibited the highest expression activity in 30-d-after-bloom fruit during whole-fruit development, and the relative value of this expression was 49.36. With fruit enlargement, the expression of the *PpEIN3a* gene gradually decreased to a relatively low level (e.g., relative value of 9.60) at 120 d after full bloom fruit. With fruit ripening, the expression of the *PpEIN3a* gene was nearly constant (e.g., 145 d after full bloom to 5 d after harvest). Interestingly, during fruit senescence, the expression of the *PpEIN3a* gene increased to its highest level (e.g., a relative value of 30.34) at 10 d after harvest fruit during fruit ripening and senescence. These results suggested that the *PpEIN3a* gene might play crucial roles during pear fruit senescence.

### 3.4 Transcript levels of *PpEIN3a* in response to multiple hormones and glucose treatments

**Figure 4:**
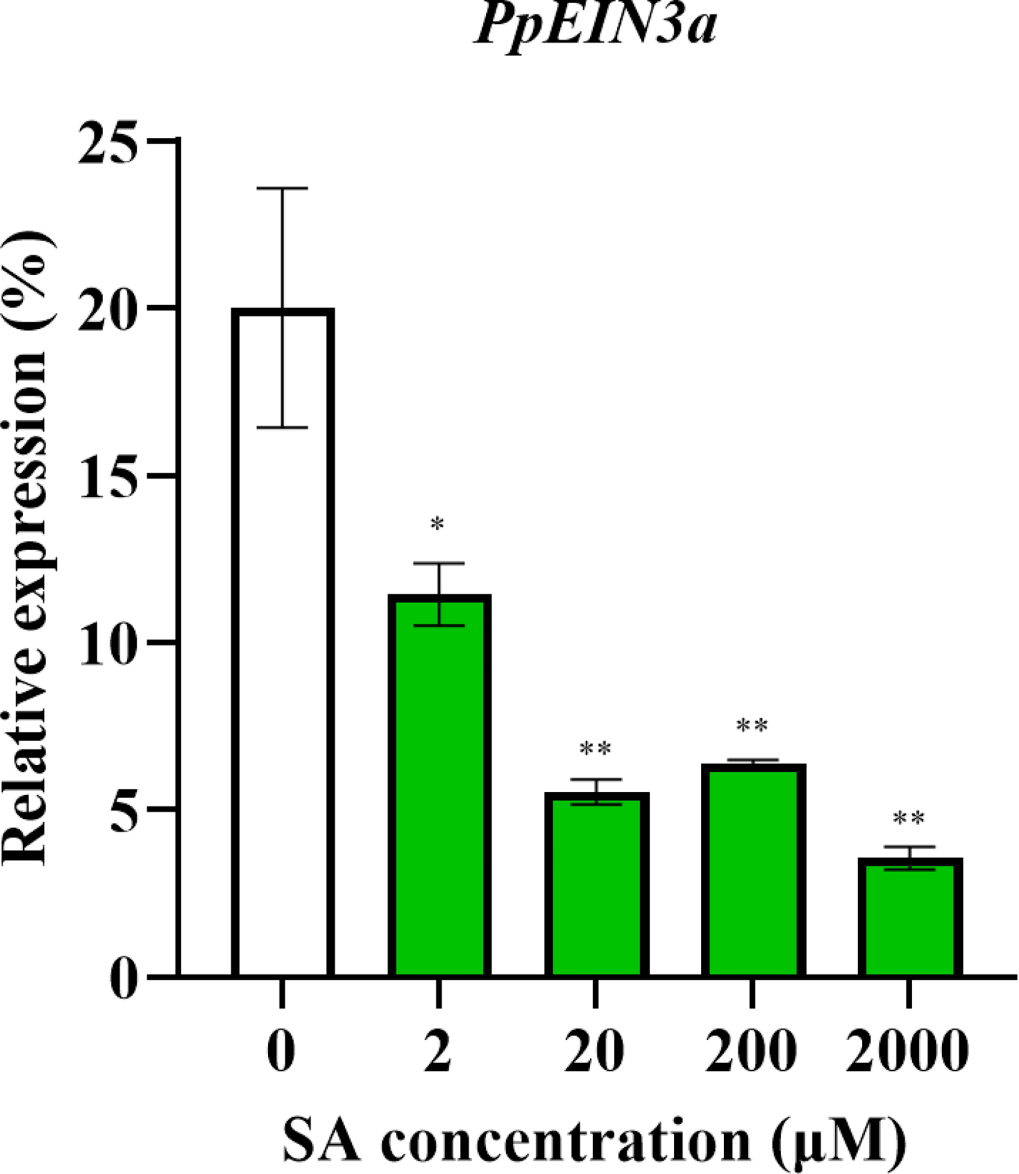
Real-time quantitative PCR analyses of the *PpEIN3a* expressions in the pulp of fruit under SA treatment for 12 h. The relative *PpEIN3a* expression values in pulp one week after fruit harvest that were treated with 0, 2, 20, 200, and 2000 μM SA for 12 h are shown as percentages of *actin* expression. The mean values and standard errors (bar) shown are from three independent experiments. Independent *t* tests for equality of means demonstrated significant (*p value < 0.05) or very significant (**p value < 0.01) differences between the control and treated pulp of fruit.

The above results showed that the *PpEIN3a* expressions were regulated during fruit senescence. SA is an important plant hormone that delays sand pear fruit senescence (Hassan et al., 2007). Therefore, SA treatments were performed to discover whether SA regulated *PpEIN3a* expression. As shown in **Figure 4**, *PpEIN3a* expression was significantly downregulated by all SA concentrations. Moreover, there were very significant differences between the control and 20/200/2000 μM SA groups.

**Figure 5:**
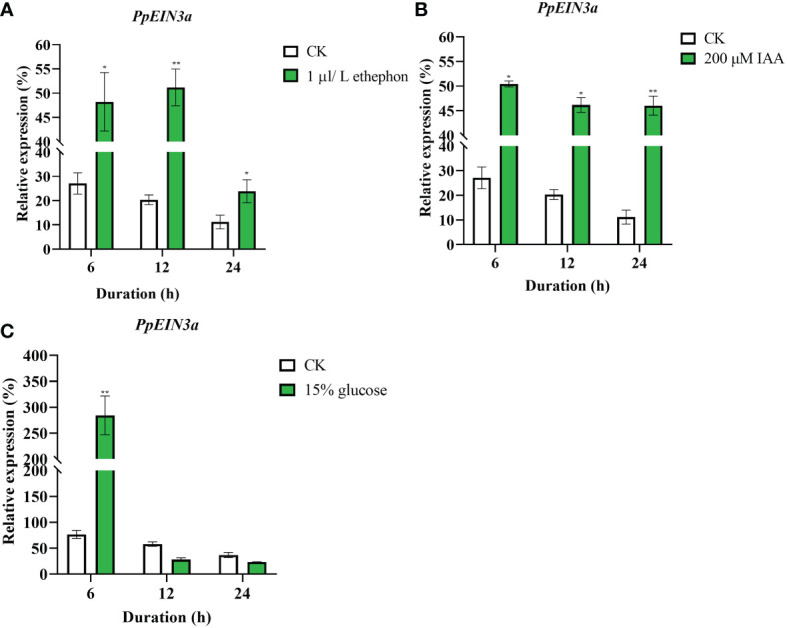
Real-time quantitative PCR analyses of the *PpEIN3a* expressions in the pulp of fruit under ethephon **(A)**, IAA **(B)** and glucose **(C)** treatments. The relative values of the *PpEIN3a* expressions in fruit pulp at one week after harvest that were treated with 1 μL/L ethephon, 200 μM IAA and 15% glucose are shown as the percentages of *actin* expression. The mean values and standard errors (bar) shown are from three independent experiments. A single asterisk shows a significant difference (*p value < 0.05), and a double asterisk represents a very significant difference (**p value < 0.01) between the treated and control pulp of fruit by *t* test.

To explore the regulation patterns of *PpEIN3a* by ethylene, auxin, and glucose, the pulp from sand pear fruit at one week after harvest was treated with 1 μL/L ethephon, 200 μM IAA, and 15% glucose. The real-time quantitative PCR results showed that the transcript accumulations of *PpEIN3a* were significantly induced by the 1 μL/L ethephon treatment at 6, 12, and 24 h (**Figure 5A**). The *PpEIN3a* expressions were also markedly upregulated by the 200 μM IAA treatment at 6, 12, and 24 h (**Figure 5B**). Interestingly, the expression of *PpEIN3a* was induced by the 15% glucose treatment at 3 and 6 h (**Figure 5C**). These results showed that SA played an antagonistic role toward ET, IAA, and glucose in regulating the expression of *PpEIN3a* to delay pear fruit senescence.

### 3.5 SA delayed shelf-life sand pear fruit senescence

**Figure 6:**
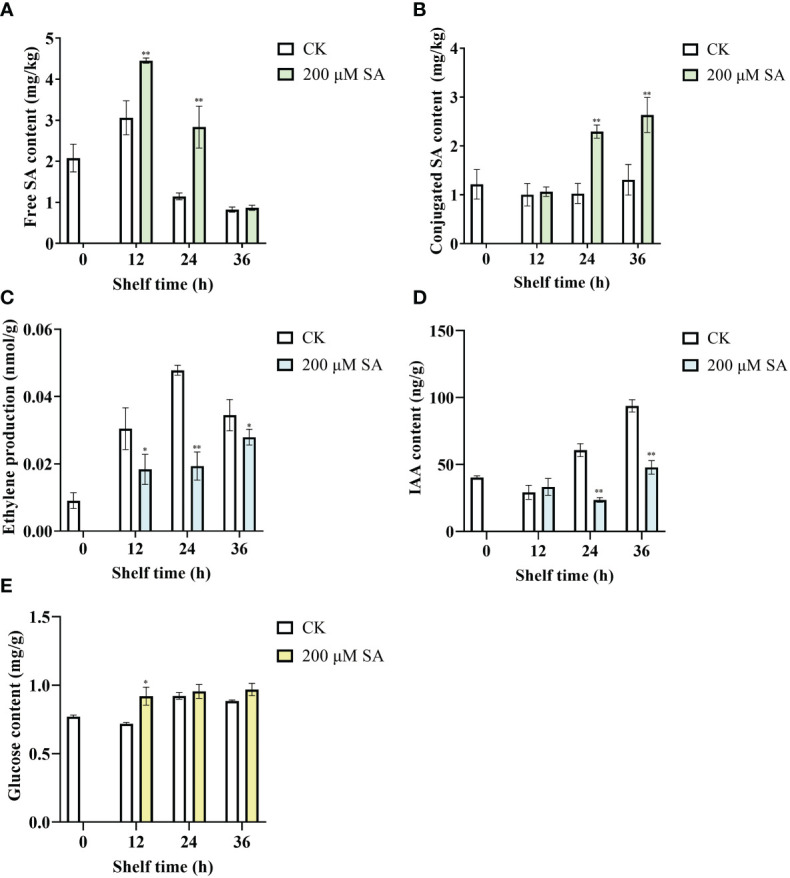
Free SA **(A)**, conjugated SA **(B)**, ethylene production **(C)**, IAA **(D)**, and Glucose **(E)** in pear fruit after SA treatment at room temperature. The mean values and standard errors (bar) from three independent experiments are shown. Independent *t* tests for equality of means demonstrated significant (*p value < 0.05) or very significant (**p value < 0.01) differences between the control and treated fruit.

Free SA contents in control fruit increased at shelf life of 12 h and then decreased, and in SA-treated fruit increased at shelf life of 12 h and 24 h, while decreased at shelf life of 36 h. However, compared with the controls, the contents of free SA in sand pear obviously increased after SA treatment at shelf life of 12 h and 24 h (**Figure 6A**). Moreover, there was no obvious change on conjugated SA contents of control fruit during shelf life, while the conjugated SA contents in sand pear significantly increased after SA treatment at shelf life of 24 h and 36 h (**Figure 6B**). The results indicated SA could induce the free and conjugated SA contents in sand pear during shelf life.

As shown in **Figure 6C**, ethylene production increased at shelf life of 12 h, 24 h, and 36 h in both control and SA-treated fruits. However, the ethylene production was significantly decreased in SA-treated fruits compared with that in controls during all the shelf life (**Figure 6C**). IAA contents of control fruit increased during the shelf life at 24 h and 36 h. While the content of IAA dramatically decreased after SA treatment compared with control at the same shelf-life time points, 24 h and 36 h (**Figure 6D**). The results showed that SA inhibited the increase in ET and IAA content of sand pear fruit. Interestingly, the contents of glucose in control fruit increased during the late shelf-life (24 h and 36 h), and in SA-treated fruit increased during all the shelf life. Moreover, the content of glucose increased when fruit were treated by SA for 12 h and then there were no differences between SA treatment and control fruit during the shelf life (**Figure 6E**). These results showed that SA played an antagonistic role toward ET and IAA during fruit senescence.

**Figure 7:**
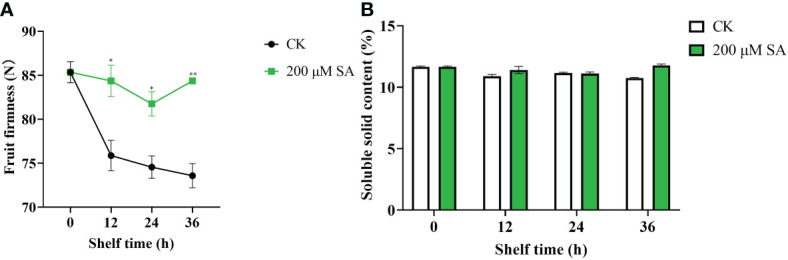
Fruit firmness **(A)** and soluble solids **(B)** contents in pear fruit after SA treatment at room temperature. The mean values and standard errors (bar) from three independent experiments are shown. Independent *t* tests for equality of means demonstrated significant (*p value < 0.05) or very significant (**p value < 0.01) differences between the control and treated fruit.

Additionally, sand pear fruit firmness in both the control and SA-treated samples showed a downward trend with longer shelf times. However, the SA-treated fruit maintained much higher fruit firmness than the controls (**Figure 7A**). This result showed that SA delayed the decrease in shelf-life sand pear fruit firmness to make fruit fresh. Moreover, the soluble solids content (SSC) in both the control and SA-treated fruit remained identical (**Figure 7B**), which suggested that the fruit quality was maintained in the SA-treated fruits.

## 4 Discussion

Recently, numerous *EIN3/EIL* genes were identified and characterized from fruit crops. For example, an *EIN3* gene was cloned from peach (*Prunus persica*) and designated as *PpEIN3*. The peach *PpEIN3* gene encodes a protein with 624 amino acids that contains a conserved EIN3 domain and a highly conserved N-terminal region (Zhang et al., 2019). A sand pear *PpEIN3b* was identified from ‘Whangkeumbae’. The deduced PpEIN3b contained a conserved EIN3 domain (Shi et al., 2019). Three *EIN3* genes (e.g., *CpEIN3a*, *CpEIN3b*, and *CpEIL1*) were also isolated from papaya fruit (Zou et al., 2020). In this study, PpEIN3a shares a relatively high homology (97.52% identity) with white pear PbEIL (*Pyrus x bretschneideri*, XP_009355060) at the amino acid level. It has 89.07% identity with PpEIN3b (*Pyrus pyrifolia*, KT726839). Nevertheless, the evolutionary relationship analysis results showed that PpEIN3a and PpEIN3b belonged to the same subgroup tree (**Figure 2**). All of the above-mentioned EIN3 proteins contain conserved EIN3 domains (**Figure 1**).

Most fruit *EIN3/EIL* genes are regulated during fruit ripening. In peach, the expression level of *PpEIN3* during late fruit development was higher than that during early fruit development. Additionally, a transgenic tomato fruit of *PpEIN3* showed early maturation, which revealed that the *EIN3* gene promoted fruit ripening (Zhang et al., 2019). The papaya *EIN3/EIL* genes were also expressed during fruit ripening. The results showed that the three *EIN3/EIL* genes were expressed at high levels in the third month (Zou et al., 2020). A previous study showed that the expression of sand pear *PpEIN3b* was regulated during fruit ripening and senescence (Shi et al., 2019). *EIL* genes of kiwifruit were involved in the regulation of fruit ripening (Yin et al., 2010). These results support the concept that *EIN3/EILs* play a role in regulating fruit ripening. Here, the *PpEIN3a* gene displayed a tissue-preferential expression pattern, and the *PpEIN3a* transcript preferentially accumulated in pear anthers (**Figure 3A**). Furthermore, the expression of the *PpEIN3a* gene was developmentally regulated during fruit senescence (**Figure 3B**), which suggested that EIN3 might be crucial in fruit senescence because of its own unique amino acid residues (**Figure 1**).

Fruit *EIN3/EIL* gene expressions are regulated by hormones and other signals during fruit ripening and senescence. For example, *MA-EIL2* is a banana fruit ripening and ethylene-inducing gene (Mbe´guie´-A-Mbe´guie´ et al., 2008). In *Arabidopsis thaliana*, although EIN3 was transcribed in both endogenous and transgenes, EIN3-flag was detected only in ACC treatment, suggesting that ethylene positively regulated EIN3 (Yanagisawa et al., 2003). Exogenous ethylene promoted the expression of *PpEIL1* and *PpEIL2* in peach fruit, and the expression levels of both tended to increase with extension of treatment time. However, 1-MCP treatment inhibited the expression of *PpEILs* (Zhao, 2009). Under exogenous ethylene treatments, papaya *CpEIN3b* and *CpEIL1* were negative regulators during fruit ripening, while *CpEIN3a* was a positive regulator. Under 1-MCP treatments, *CpEIN3a* was negatively correlated with respiration rates and ethylene production (Zou et al., 2020). Additionally, papaya CpEIL1 is involved in regulating fruit ripening by interacting with the auxin response factor (Zhang et al., 2020b), which suggests that *CpEIL1* expression might be regulated by the crosstalk of ethylene and auxin. Pear *PpEIN3b* expression was inhibited by SA, glucose, and disease but was induced by ACC during sand pear fruit ripening and senescence, which suggested that pear *PpEIN3b* might be a negative regulator in delaying fruit ripening and senescence (Shi et al., 2019). Yue et al. (2020) showed auxin induced ET biosynthesis in apple fruit through activation of *MdARF5* expression. EIN3 proteins are transcription factors and the key regulators of ET signaling that sustain a variety of plant responses to ET. Deng et al. (2022) reported that Mediator (MED) subunit MED25 in tomato regulated the expression of ripening genes by interacting with EILs transcription factors to link ethylene signaling with Pol II mechanism. In the present study, *PpEIN3a* expression was inhibited by SA (**Figure 4**) but was induced by ethylene, auxin, and glucose (**Figure 5**), which suggested that PpEIN3a might be a positive regulator during pear fruit senescence. Glucose inhibited EIN3 stability (Yanagisawa et al., 2003), which may explain why pear *PpEIN3b* expression was reduced after glucose treatments (Shi et al., 2019), while glucose induced *PpEIN3a* expression in this study, which may be due to the particular structure of PpEIN3a including the substitutions and deletions, which might lead to its special function during fruit ripening and senescence (**Supplementary Figure 1**). This might also be the reason that leads to the relatively distant evolutionary relationship between PpEIN3a and PpEIN3b. Also, there were no significant different *PpEIN3a* expression levels in diseased fruit compared with control (**Supplementary Figure 2**), while *PpEIN3b* expression was dramatically inhibited by disease during sand pear fruit senescence (Shi et al., 2019). Peach *PpEIN3* was overexpressed in tomato, and the transgenic tomato fruit reddened earlier than the control fruit, while fruit ripening was inhibited upon the knockdown of *PpEIN3*, which showed that *PpEIN3* accelerated fruit ripening and suggested that PpEIN3 might be a positive regulator during fruit ripening (Zhang et al., 2019). Therefore, *PpEIN3a* may be a positive regulator during pear fruit ripening and senescence, similar to peach *PpEIN3* (Zhang et al., 2019). Xu et al. (2021) showed that DcEIL3-1 interacted with DcWRKY75, a homolog of ethylene signaling core transcription factor EIN3 in *Arabidopsis thaliana*. Silencing *DcEIL3-1* can delay the senescence of carnation petals.

The physiological mechanism of SA delaying fruit ripening and senescence has been studied and is summarized as follows: Leslie and Romani (1988) found that SA can inhibit the biosynthesis of ET from ACC. The final step of the ET biosynthetic pathway is that ACC oxidase (ACO) converts ACC to ET. Furthermore, SA inhibits ET biosynthesis by inhibiting the activity of ACO (Fan et al., 1996). Srivastava and Dwivedi (2000) explored that SA delayed banana fruit ripening (Musa acuminata) by decreasing fruit softening, pulp:peel ratios, reducing sugar contents, invertase, respiration rates, and the activities of major cell wall-degrading enzymes (e.g., cellulase, polygalacturonase and xylanase)/the major enzymatic antioxidants (e.g., catalase and peroxidase). Treatment with SA, acetylsalicylic acid (ASA) and oxalic acid (OA) delayed the postharvest ripening process of sweet cherry cultivars harvested at commercial maturity stage, which showed decreased acidity, color change, hardness, and maintained quality attributes for a longer time than the control group (Valero et al., 2011). In addition, SA can maintain fruit quality for longer periods by continuously increasing total phenolics, anthocyanins and antioxidant activities during storage (Srivastava and Dwivedi, 2000). SA delayed the senescence of sand pear fruit by enhancing the activity of superoxide dismutase/peroxidase enzymes, reducing malondialdehyde contents, and decreasing water loss ratios (Hassan et al., 2007). SA also reduced sand pear fruit decay and tissue browning, and maintained the postharvest quality parameters of pear up to 60 days of cold storage (Adhikary et al., 2021). In this study, SA treatment delayed the decrease in post-harvested sand pear fruit firmness (**Figure 7A**). The contents of free SA (**Figure 6A**) and conjugated SA (**Figure 6B**) in sand pear increased after SA treatment. Moreover, the content of ethylene (**Figure 6C**) and auxin (IAA, **Figure 6D**) decreased after SA treatment compared with control during fruit senescence. Additionally, glucose content (**Figure 6E**) and SSC (**Figure 7B**) during the shelf life were maintained after SA treatment. The results indicated that SA could delay fruit senescence and maintain fruit quality. However, the molecular mechanism by which SA delays fruit senescence remains unclear and needs much deeper study. Here, the *EIN3* gene, as a crucial element of the ET signaling pathway, will be further studied to resolve the above molecular mechanism.

## 5 Conclusions

**Figure 8:**
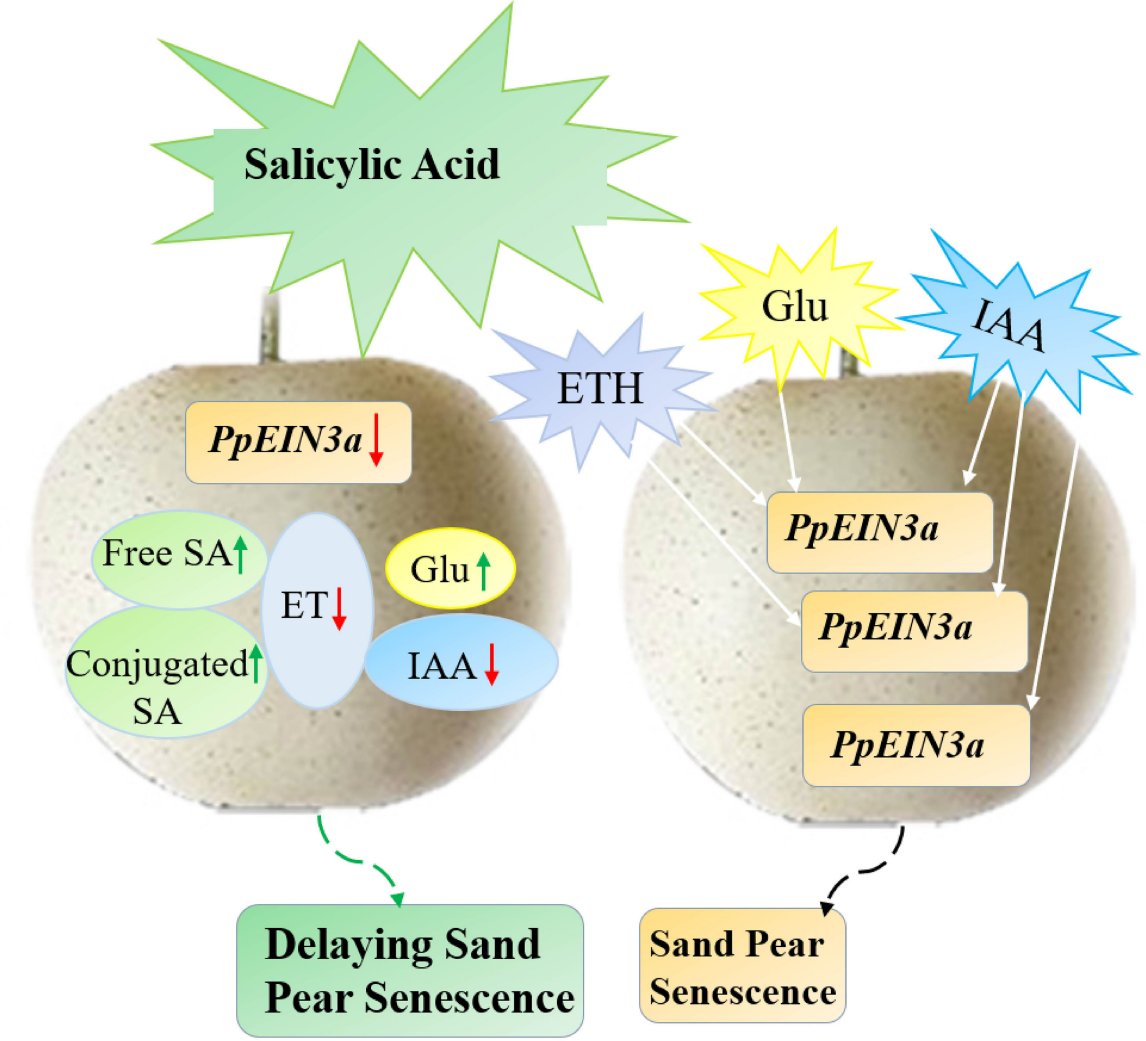
The model pattern of salicylic acid (SA)-mediated sand pear fruit senescence *via* regulating *PpEIN3a* expression. SA may delay fruit senescence by influencing the contents of endogenous SA, ET, IAA, and Glu that regulating *PpEIN3a* expression in pulp. Green and white arrows represent up-regulation, and red arrows represent down-regulation.

In this study, we concluded a model that SA played an antagonistic role toward ET, IAA, and glucose in regulating the expression of *PpEIN3a* to delay pear fruit senescence (**Figure 8**). This study presented that SA increased endogenous SA levels but reduced the content of endogenous ET and IAA in sand pear, while glucose content increased after SA treatment during early shelf life. However, there was no influence for glucose content after SA treatment during late shelf life, connecting with the results that SA delayed the decrease in sand pear fruit firmness and the SSC was maintained in between the SA-treated and control fruit, which suggested that SA might maintain the sand pear quality and it could be an effective preservative for climacteric fleshy fruit. It also suggests that plant hormones, including SA, ET, auxin and other signals, such as glucose, interact to control fruit senescence by regulating senescence-related genes.

## Data availability statement

The original contributions presented in the study are publicly available. This data can be found here: NCBI, KT726838.

## Author contributions

HS conceived this study. HS, YX, LH, KZ, and YL performed the experiments. HS, YX and LH analyzed the data. HS, YX and LH wrote the manuscript. KZ, YL, XZ, HW and WW revised the manuscript. All authors contributed to the article and approved the submitted version.
