# Supplementary material for: Salicylic acid delays pear fruit senescence by playing an antagonistic role toward ethylene, auxin, and glucose in regulating the expression of PpEIN3a
Source: Front Plant Sci. 2023 Jan 11;13:1096645. doi: 10.3389/fpls.2022.1096645 (PMC9875596; doi:10.3389/fpls.2022.1096645)
Supplement: Supplementary file 1 [file DataSheet_1.docx]

PpEIN3a MGIFEEMGFCGNLDFLSAPSGEGEAAPEHEPEATGEEDYSDEEMDVDELERRMWRDRMLL 60

PpEIN3b MGIFEELGFSGNLDYLLAPSGEGDAAPEHEQEATVEEDYSDDETDVDELEKRMWRDRMLL 60

PpEIN3a K-RLKEQTKGKERVDNARQRQSQEQARRKKMSRAQDGILKYMLKMMEVCKAQGFVYGIIP 119

PpEIN3b KKKLKEQTKGKEGVDNARQCQSQEQARRKKMSRAQDGILKYMLKMMEVCKAQGFVYGIIP 120

PpEIN3a EKGKPVSGASDNLRAWWKEKVRFDRNGPAAISKYQADHSIPGKNEDCSVVASTPHTLQEL 179

PpEIN3b EKGKPVSGASDNLRAWWKEKVRFDRNGPAAISKYQADHSIPGKNEDCSVVASTPHTLQEL 180

PpEIN3a QDTTLGSLLSALMQHCDPPQRRFPLEKGVAPPWWPTGNEEWWPQLNLPKDQGPPPYKKPH 239

PpEIN3b QDTTLGSLLSALMQHCDPPQRRFPLEKGVAPPWWPTGNEDWWPQLNLPKDQGPPPYKKPH 240

PpEIN3a DLKKAWKVGVLTAVIKHMSPDIAKIRKLVRQSKCLQDKMTAKESATWLAIINQEEALARR 299

PpEIN3b DLKKAWKVGVLTAVIKHMSPDIAKIRKLVRQSKCLQDKMTAKESATWLAIINQEEALARR 300

PpEIN3a LYPDRCPPPLAGGGGSLAISGTSDYDVEGVDDDENVETEDCKPLVNHFNIGTAGQRER-L 358

PpEIN3b LYPDRCPPPFAGGSESVAISGTSDYDVEGVDDDENIEIEDCKPLVNHFNIGATGQRERQV 360

PpEIN3a VPQIKGELIEINSDFGQKRKQLSEEPQMMLNQKIYTCEYPQCPYHDYRLGFLNITARNNH 418

PpEIN3b LPQVKGELIEINSDFGPKRKQLAEEPQMMLDQKYYTCEYLQCPYHDYRLGFLDITARNNH 420

PpEIN3a QMNCQYRSNSSQVFGMSSFQLHNEKSVGFSLPIAQPPAPTFQQSVNQASRFNASGVDDG- 477

PpEIN3b QLNCPYRNNSSQVLGMSSFQIHNEKPVGFSLPIAQPPAPTFQQSVNQASRFNASGLGLVD 480

PpEIN3a QKMISELMSFYDSNVQQNQNCNPGNLHIVENRNQQQSKYQFPMNDNFFGQGVDTGCNINM 537

PpEIN3b NGQKSELISFYDSNIQQNKNCNPANLHIVDNRNSQHSKYQFPMNDNFFGQGVDVGRNINM 540

PpEIN3a SEPAPMPMLHPGFASPEVQFDQCIAFDSPFGNNSNEDVDIRFGSPLHLAPVGYNVMDPPL 597

PpEIN3b SELAPMPMLHPGFASPEVQFDQCMAFDPPFGNNTNENVDIRFESPLHLAPVDYNVMDQPL 600

PpEIN3a SQDP—WFP 604

PpEIN3b KQDASIWFQ 609

**Supplementary Figure 1.** Sequence alignment between the pear fruit PpEIN3a and PpEIN3b proteins. Amino acid substitutions and deletions are highlighted in black. The accession numbers of the pear fruit EIN3 proteins in GenBank are KT726838 (*Pyrus pyrifolia* PpEIN3a) and KT726839 (PpEIN3b).
