# Supplementary material for: Salicylic acid delays pear fruit senescence by playing an antagonistic role toward ethylene, auxin, and glucose in regulating the expression of PpEIN3a
Source: Front Plant Sci. 2023 Jan 11;13:1096645. doi: 10.3389/fpls.2022.1096645 (PMC9875596; doi:10.3389/fpls.2022.1096645)
Supplement: Supplementary file 2 [file DataSheet_2.docx]

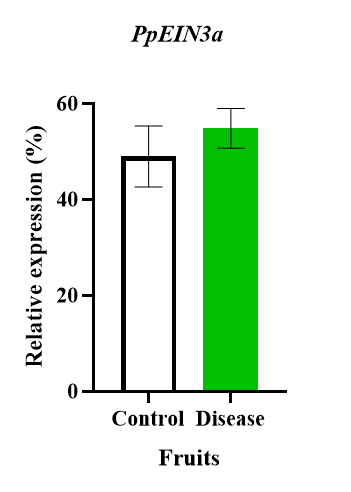


**Supplementary Figure 2.** Real-time quantitative PCR analysis of the *PpEIN3a* expression in the pulp of diseased fruit. The relative values of the *PpEIN3a* expression in 20-day-after-harvest diseased fruit pulp are shown as the percentage of actin expression. The mean values and standard errors (bar) shown are from three independent experiments.
